# Cell anatomy and network input explain differences within but not between leech touch cells at two different locations

**DOI:** 10.3389/fncel.2023.1186997

**Published:** 2023-07-25

**Authors:** Sonja Meiser, Jana Marie Sleeboom, Ihor Arkhypchuk, Kevin Sandbote, Jutta Kretzberg

**Affiliations:** ^1^Department of Neuroscience, Computational Neuroscience, Faculty VI, University of Oldenburg, Oldenburg, Germany; ^2^Institute of Physiology II, Faculty of Medicine, University Clinic Bonn (UKB), University of Bonn, Bonn, Germany; ^3^Department of Neuroscience, Cluster of Excellence Hearing4all, Faculty VI, University of Oldenburg, Oldenburg, Germany; ^4^Research Center Neurosensory Science, University of Oldenburg, Oldenburg, Germany

**Keywords:** invertebrate, mechanoreceptor, multi-compartment model, neuronal excitability, response variability, neuronal anatomy

## Abstract

Mechanosensory cells in the leech share several common features with mechanoreceptors in the human glabrous skin. Previous studies showed that the six T (touch) cells in each body segment of the leech are highly variable in their responses to somatic current injection and change their excitability over time. Here, we investigate three potential reasons for this variability in excitability by comparing the responses of T cells at two soma locations (T2 and T3): (1) Differential effects of time-dependent changes in excitability, (2) divergent synaptic input from the network, and (3) different anatomical structures. These hypotheses were explored with a combination of electrophysiological double recordings, 3D reconstruction of neurobiotin-filled cells, and compartmental model simulations. Current injection triggered significantly more spikes with shorter latency and larger amplitudes in cells at soma location T2 than at T3. During longer recordings, cells at both locations increased their excitability over time in the same way. T2 and T3 cells received the same amount of synaptic input from the unstimulated network, and the polysynaptic connections between both T cells were mutually symmetric. However, we found a striking anatomical difference: While in our data set all T2 cells innervated two roots connecting the ganglion with the skin, 50% of the T3 cells had only one root process. The sub-sample of T3 cells with one root process was significantly less excitable than the T3 cells with two root processes and the T2 cells. To test if the additional root process causes higher excitability, we simulated the responses of 3D reconstructed cells of both anatomies with detailed multi-compartment models. The anatomical subtypes do not differ in excitability when identical biophysical parameters and a homogeneous channel distribution are assumed. Hence, all three hypotheses may contribute to the highly variable T cell responses, but none of them is the only factor accounting for the observed systematic difference in excitability between cells at T2 vs. T3 soma location. Therefore, future patch clamp and modeling studies are needed to analyze how biophysical properties and spatial distribution of ion channels on the cell surface contribute to the variability and systematic differences of electrophysiological phenotypes.

## 1. Introduction

Despite the apparent necessity to react in a robust and appropriate way to sensory stimuli, variability is omnipresent in the nervous systems of vertebrates and invertebrates. On the level of a single neuron, trial-to-trial variability of the same neuron’s spike counts and spike timing to identical repeated stimulation was demonstrated in many sensory systems (Lestienne, 2001). Since neuronal activity depends on stochastic events like random channel opening and quantal transmitter release, noise is introduced by multiple factors on the sensory, cellular and network level (Faisal et al., 2008). On the sensory level, the stochastic nature of physical stimuli and their transduction process already leads to trial-by-trial variation of the input to the sensory system (Schneeweis and Schnapf, 1999; Grewe et al., 2003). Additional response variability originates from the cellular level, where channel noise causes jitter in the threshold and timing of the spike generation (Faisal et al., 2008). Additionally, the stochastic presynaptic release and postsynaptic uptake of transmitter vesicles, leads to highly variable postsynaptic responses (Ribrault et al., 2011). However, variations in neuronal responses to repeated stimulation are not necessarily caused by noise. Activity changes over time can indicate relevant shifts in neuronal information processing that allow stable reactions under variable conditions (Fontanini and Katz, 2008). In particular in sensory systems, neuronal adaptation optimizes the neuronal sensitivity to the recent environmental conditions (Clifford et al., 2007). Repeated stimulation can cause plasticity at various levels of the nervous system, from individual synapses to entire neural circuits (Feldman, 2009). Thereby, the post-synaptic responses change systematically over time, providing the basis for learning and memory (Magee and Grienberger, 2020). Moreover, in addition to the local effects of adaptation and synaptic plasticity, also global shifts in physiological states induced by neuromodulation (Marder et al., 2014) or homeostatic mechanisms (Turrigiano, 2008) impact individual cells. In this sense, the measured activity changes over time provide evidence for flexible reactions of neuronal systems to an ever-changing environment. By combining global and local mechanisms of flexibility, nervous systems maintain a stable operating regime for robust sensory information processing (Lütcke et al., 2013).

Functional diversity between cell types is the very basis for the perception of different aspects of sensory stimuli. This can be seen, e.g., in retinal ganglion cells specialized to color, contrast, or motion of sensory stimuli (Gollisch and Meister, 2010). In contrast, variations between neurons of the same type could at first glance be interpreted as an additional source of random variability. Such inter-cell type variations were found on the level of functionally relevant gene expression for neurons of the same neuroanatomical type, e.g., in brainstem neurons (Park et al., 2014) and somatosensory dorsal root ganglion cells (Zheng et al., 2019). Conversely, molecularly defined cell types exhibit morphological diversity of dendrites and axons in all brain regions of the mouse (Peng et al., 2021). Also the variability of ion channel densities on the cell surface, e.g., of pyramidal cells adds to the neuronal diversity (Nusser, 2009). In consequence, neurons of the same cell type differ considerably in their electrophysiological response characteristics, as was shown, e.g., for stellate cells in the mouse cortex (Pastoll et al., 2020), the crustacean stomatogastric ganglion (Prinz et al., 2004) and mechanosensory neurons of the leech (Scherer et al., 2022). However, despite the omnipresent variability on the cellular level, the network activity can still be stable, leading to robust performance between animals on a behavioral level (Prinz et al., 2004). This robustness in functional output was proposed to rely on functionally overlapping ion channels, that can substitute each other (Goaillard and Marder, 2021), and to be the result of tuning of cellular properties to circuit-level set points (Pastoll et al., 2020). With this perspective, neuronal phenotypes can be considered a continuum rather than as discrete categories, and providing the basis for the surprising robustness of nervous systems toward perturbations and changing environmental conditions (Goaillard and Marder, 2021).

Most vertebrate nervous systems are very large and complex (Charles and Jane, 2015) and require elaborated experimental approaches to characterize neurons at single cell level and investigate potential differences between cell subtypes (Baier and Scott, 2009). Hence, the analysis of individual cells and their neuronal flexibility is often studied in invertebrate systems (Goaillard and Marder, 2021). The medicinal leech *Hirudo verbana* proved to be a useful model organism in systems neuroscience for the investigation of sensory processing, network dynamics, and even behavioral choice (Kristan et al., 2005; Wagenaar, 2015). These topics can be studied on the level of multiple individually characterized neurons in the stereotyped, small, and experimentally easily accessible nervous system of the leech (Kristan et al., 2005; Wagenaar, 2015). Of the approximately 400 neurons in each segmental ganglion, only 14 are mechanoreceptors, i.e., six touch (T), four pressure (P), and four nociceptive (N) cells, named after their preferred intensity ranges of tactile stimuli (Nicholls and Baylor, 1968; Kristan et al., 2005). These cell types can be easily distinguished based on their responses to somatic current injection, even in isolated ganglia without the skin attached (Nicholls and Baylor, 1968), and feature strong similarities with mechanoreceptors in vertebrates (Burrell, 2017).

In this study, we focus on the most sensitive of the leech mechanoreceptors, the T cells, whose responses resemble the activity of rapidly adapting afferents in mammals (Burrell, 2017). T cells respond to light touch to the skin and encode the velocity of tactile stimuli and changes in pressure (Carlton and McVean, 1995, 1996; Kretzberg et al., 2016) within the overlapping receptive fields they innervate with their extended processes (Blackshaw, 1981). Additionally, it is known that T cells receive synaptic inputs via polysynaptic connects from the other mechanoreceptor types (Baylor and Nicholls, 1969a; Burgin and Szczupak, 2003) and are mutually coupled via electrical and polysynaptic connections (Baylor and Nicholls, 1969b; Gu et al., 1989). The cell bodies of the six T cells within each ganglion are located at distinct bilateral positions in the central ganglion, named T1, T2, and T3 (Tomina and Wagenaar, 2017). For brevity, we call a T cell with soma location T3 “T3 cell,” and analogously for T2 and T1. The three bilateral pairs of T cells within one ganglion have their receptive fields either in the dorsal, lateral, or ventral skin area on one side of the body wall. These receptive fields correspond to the T cell anatomy. T cells with a dorsal receptive field have only one root process toward the direction of the roots, whereas lateral and ventral T cells develop two root processes (Nicholls and Baylor, 1968). However, T cells with a ventral receptive field were found at variable soma locations between preparations (Kretzberg et al., 2016), making it hard to distinguish their innervations from their locations in the ganglion. For the investigation of electrophysiological properties, the three pairs of T cells were considered as one homogeneous cell type that has action potentials up to 70 mV in amplitude and about 2 ms in duration and tends to fire in bursts (Nicholls and Baylor, 1968).

A recent study investigated how variable neuronal response features of T cells are in comparison to two other well-known cell types of the leech—over time and between cells (Scherer et al., 2022). The study revealed that the initial responses of T cells to identical stimulation cover a wide range from 0 to 23 spikes, with high temporal precision of short latencies between 5 and 10 ms. In contrast, the neurosecretory Retzius cells elicited consistently 2 to 5 spikes with variable, long latencies of 10 to 70 ms. A systematic increase of excitability over recording time was found in the mechanosensory T and P cells, but not in Retzius cells. This change in excitability could contribute to the response variability of the two mechanosensory cell types. Comparing the results of Scherer et al. (2022), which were recorded exclusively from T3 cells, with the previous study of Meiser et al. (2019), that pooled T cells at all three soma locations with a high percentage of T2 cells, noticeable differences become evident. Meiser et al. (2019) reported a higher initial spike count (SC) with a median of 14.5 compared to 4 spikes in 500 ms, and a stronger increase in excitability over recording time than Scherer et al. (2022). Therefore, we hypothesize that the divergent results of both studies could originate from systematic differences between the responses of T cells at different soma locations.

Following up on these previous studies, we here systematically compare the responses to current injection in double recordings of T cells at soma locations T2 and T3. Consistent with Meiser et al. (2019) and Scherer et al. (2022), we find high variability in excitability within and additionally systematic differences between both locations. Applying electrophysiological double recordings, dye injections for anatomical studies, and compartmental modeling, we investigate three potential factors for the observed response variability within and between T cell locations: (1) The time-dependent activity changes reported by Meiser et al. (2019) and Scherer et al. (2022). (2) Synaptic input that the T cells receive from the unstimulated network (Baylor and Nicholls, 1969b; Scherer et al., 2022), and from a coupled T cell in the same ganglion (Baylor and Nicholls, 1969b; Meiser et al., 2019) (3) The anatomical structure (Muller and McMahan, 1976; Segura et al., 2020), which we also compare to T1 cells. While all of them contribute to the variability within the T cells recorded at each of the soma locations, we rule out that any of these factors can exclusively explain the systematic difference in excitability between T2 and T3 cells.

## 2. Materials and methods

Experiments (including intracellular double recordings and neurobiotin staining) were performed on in total 240 neurons in 117 ganglia from 90 preparations of adult hermaphrodite medicinal leeches (see Supplementary Table 1 for details) (*Hirudo verbana*; *Biebertaler Leech Breeding Farm*, 35444 Biebertal, Germany). According to German regulations, no approval of an ethics committee is needed for the work on these invertebrates.

**FIGURE 1:**
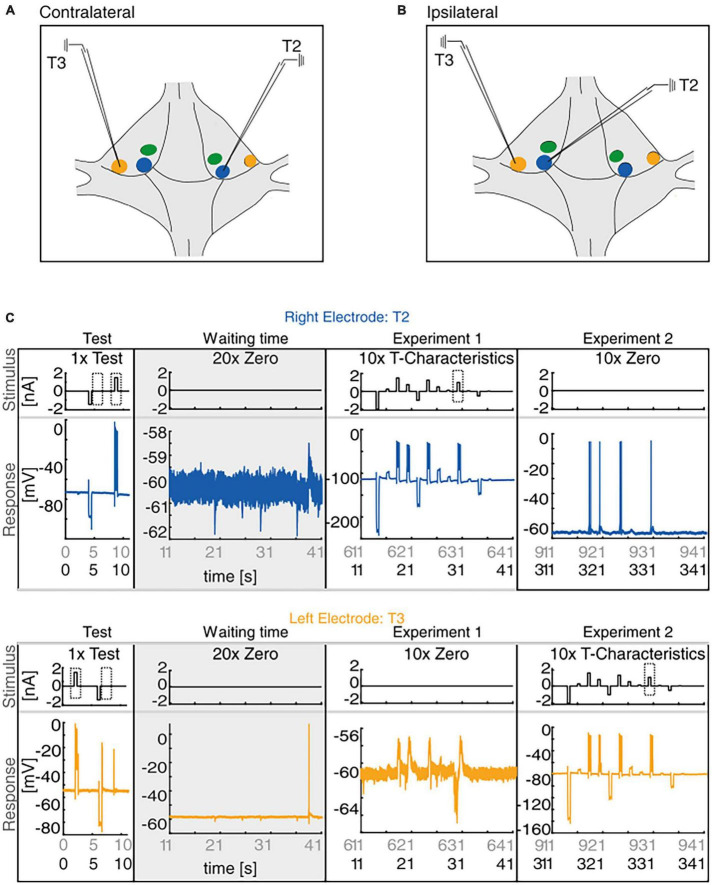
Experimental Design. **(A,B)** Sketch of the isolated ganglion. The membrane potential of the T cells with their somata located at T1 (green), at T2 (blue), and at T3 (yellow) locations which were recorded simultaneously with intracellular electrodes either in contralateral **(A)** or ipsilateral **(B)** configuration. **(C)** The main experiment comprised three different stimulus protocols. After inserting the electrodes into the target cells the test stimulus was applied once for 11 s, consisting of a positive and a negative current pulse that were injected alternately into both cells. After the test protocol, we recorded in half of our experiments the membrane potential of both cells during 10 min of waiting time (gray) to analyze spontaneous synaptic inputs and spiking activity (please note the different scale of the *y*-axes scaling for recordings with and without spikes). After this waiting period, we first applied via the right electrode the stimulation protocol “T-Characteristics” 10 times into the cell soma (Experiment 1, here T2), and then repeated this stimulation with the left electrode (Experiment 2, here T3). In the other half of the experiments the same stimulation protocols were injected in the same sequence without the waiting time between test and stimulation protocols (black labels on time axes in panels Experiment 1 and Experiment 2). The dashed boxes indicate the stimulation periods during the test protocol and the “T-Characteristics” protocol that were analyzed in detail.

The leeches were kept under natural day-light-cycles, at room temperature in 24 L tanks filled with artificial pond water (ocean sea salt diluted with purified water, 1:1000). The animals were anesthetized in ice-cold water for approximately 20 min before and during dissection in ice-cold leech saline [115 mM sodium chloride, 4 mM potassium chloride, 1.8 mM calcium chloride, 10 mM Glucose, 4.6 mM Tris maleate, 5.4 mM Tris base; pH 7.4 (Muller and Scott, 1981)]. We used isolated midbody ganglia, dissected from segments 9–12 for intracellular double recordings and from segments 7–16 for the intracellular dye injections. They were pinned, ventral side up, to a plastic Petri dish, coated with the silicone elastomer Sylgard (Dow Corning Corporation, Midland, MI, United States). To avoid potential effects of experimental stimulation history, we limited the recording to one experiment on one pair of T cells per ganglion (Figure 1).

### 2.1. Intracellular electrophysiology

We performed intracellular double recordings in the current clamp mode from *N* = 82 pairs of mechanosensory T2 and T3 cells in ipsilateral (*n* = 43) or in contralateral (*n* = 39) recording configuration (Figures 1A, B, see Supplementary Table 1 for details on sample sizes). For this purpose, sharp microelectrodes were pulled from borosilicate glass (TW100F-4, World Precision Instruments Inc., Sarasota, FL, United States) with a P97 Flaming Brown micropipette puller (Sutter Instruments Company, Novato, CA, United States) and filled with 4 M potassium acetate (pH adjusted to 7.4). These electrodes used for the intracellular double recordings had resistances ranging from 11 to 31 MΩ ($\tilde{x}$ = 21.5 MΩ, Q_1_ = 17 MΩ, Q_3_ = 26 MΩ) and were held by two mechanical micromanipulators type MX-1 (TR 1, Narishige, Tokyo, Japan). BA-1s amplifiers (NPI Electronic, Tamm, Germany) connected the electrodes via an interface BNC-2090 with the NI PCI-6036E board (National Instruments, Austin, TX, USA) to the computer. We used custom-written MATLAB software (MATLAB 2021b, MathWorks, Natick, MA, United States) to inject current into the somata of the T cells and record at the same time their membrane potential (sample rate 10 kHz).

Additionally, a digital multimeter (PeakTech 2025, Ahrensburg, Germany) tracked the temperature in the Petri dish during the T cell-T cell double recordings. Room temperature ranged from 18 to 22°C. We never recorded a temperature change of more than 2°C during the same experiment.

### 2.2. Experimental design of the intracellular double recordings

For the intracellular double recordings, we inserted each electrode into one of the two target T cells at soma locations T2 and at T3, either in contralateral (opposite sides of the ganglion) or ipsilateral (same side of the ganglion) configuration (Figures 1A, B). Cells were identified according to their soma location by using the ganglion map of Tomina and Wagenaar (2017) and by comparison of the recorded spike responses to the standard T cell responses as shown in Nicholls and Baylor (1968).

Our experiment consisted of up to three different stimulus protocols (Figure 1) and lasted either 641 or 1241 s. First, the 11-s-long test protocol was applied once with alternating current injection to both cells (Figure 1C, left column), which consisted of one 500 ms long negative current pulse of −1.5 nA amplitude, and one 500 ms long positive current pulse of +1.5 nA amplitude. Via the right electrode, the negative pulse was applied at second 4 and the positive pulse at second 8.5 of the test stimulus. For the left electrode, the positive pulse started at second 2 and the negative pulse at second 6. This enabled us to record the initial pre- and postsynaptic response of both T cells, without being affected by massive previous stimulation. In 40 of our *N* = 82 recordings the right electrode was inserted into the T2 cell and the left one into the T3 cell (see example in Figure 1), and in 42 recordings vice versa. In 42 of the 82 recordings the test protocol was followed by 10 min of waiting time (20 trials of 30 s) without electrical stimulation (Figure 1C, gray column). This unstimulated period allowed us to analyze spontaneous synaptic inputs from the network to the T cells.

The stimulus protocol “T-Characteristics” (Figure 1C, third column), developed by Meiser et al. (2019), had a total length of 30 s and consisted of 12 pseudo-randomized current pulses in a fixed order, varying in amplitude from −2 to + 1.5 nA, each with a duration of 500 ms. The pulses were separated by 1500 ms without current input (Meiser et al., 2019; Scherer et al., 2022). In all recordings, the “T-Characteristics” stimulus protocol was first injected via the right electrode, while the other T cell on the ipsilateral or the contralateral side of the ganglion remained unstimulated (Figure 1C, third column). Afterward, the exact same stimulation was applied via the left electrode (Figure 1C, fourth column). Consequently, the stimulation with the “T-Characteristics”- protocol started for each of the T cells at one of four different time points, depending on whether the experiment included the waiting time (gray area labeling in the horizontal axis of Figure 1C) or not (black time labeling in Figure 1C) and recording with the left vs. right electrode. The first two stimulation starting times were applied to *n* = 20 T2 and *n* = 20 T3 cells, the last two stimulation starting times were applied to *n* = 22 T2 and *n* = 22 T3 cells.

### 2.3. Data analysis of the intracellular double recordings

For pre-processing, we applied a notch filter to the recorded membrane potential. The filter removed half the power of the frequency components in the range of 47–53 Hz to reduce the power-line hum. The notch filtered traces were then smoothed with the MATLAB *movmean*-function with a sliding window of 10 ms. We further corrected all membrane potential values for each time point for their individual, mostly negative electrode offset ($\tilde{x}$ = −4 mV, Q_1_ = −7 mV, Q_3_ = −1 mV) measured at the end of the experiment, by assuming a linear electrode drift over the total recording time.

**FIGURE 2:**
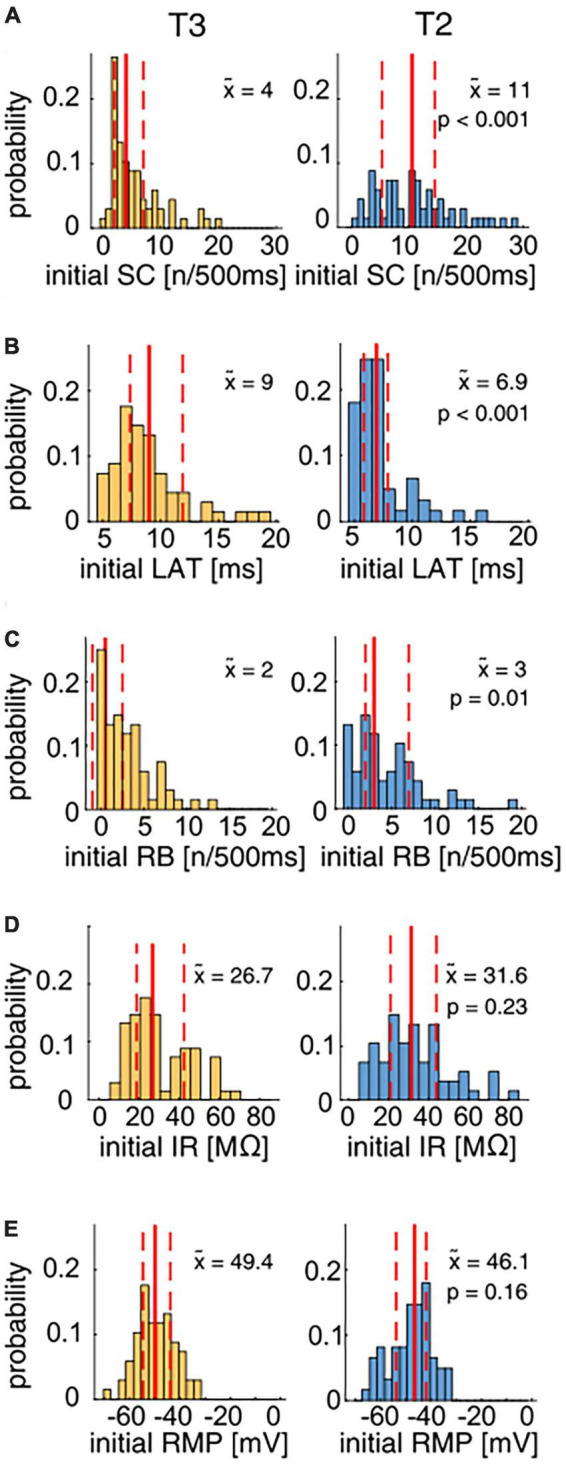
Physiological parameter distributions for T2 and T3 cells in response to the test protocol. The distributions of the physiological parameters spike count (SC), latency (LAT), rebound spikes (RB), input resistance (IR), and resting membrane potential (RMP) of T3 (yellow, *N* = 68) and T2 (blue, *N* = 61) cells were compared using the non-parametric Wilcoxon rank sum test (see *p*-values in the panels). Median values ($\tilde{x}$) of the distributions obtained for cells at each location are given by solid red lines and numbers in the panels, the quartiles of the distributions are indicated by dashed red lines. **(A)** The initial SC distribution shows the high variability for both T2 and T3 cells. The median SC for T2 cells (blue) was significantly higher than for T3 cells (yellow). **(B)** The response latency distribution is skewed for both T cell types. T2 cells responded significantly faster to a 1.5 nA current pulse than T3 cells. **(C)** The distributions of the number of rebound spikes after a hyperpolarizing current step reach from 0 to > 10 for both subtypes. T2 cells fired significantly more rebound spikes than T3 cells. **(D)** The input resistances were highly variable for both types and did not differ significantly between T2 and T3. **(E)** The initial membrane potential ranged approximately from –65 to –35 mV for T cells on both soma locations, without a significant difference.

**FIGURE 3:**
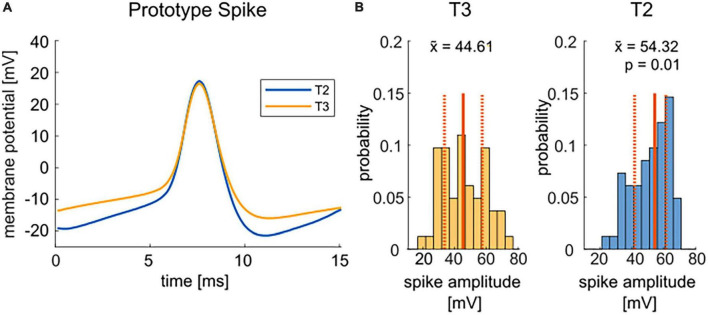
Comparison of T2 and T3 cell spike shapes. **(A)** Mean spike shapes of the second spike in response to the test pulse of +1.5 nA current injection have a more negative afterhyperpolarization for T2 spikes (blue, average of *n* = 54) than T3 cell spikes (yellow, average of *n* = 59). The mean was calculated for all cells that responded with at least two spikes to the test pulse. **(B)** The distributions of the absolute spike height (| V_*max*_–V_*min*_|) for T2 and T3 cells are significantly different (*p* = 0.01, non-parametric Wilcoxon rank sum test). Median values ($\tilde{x}$) are given by solid red lines and numbers in the panels, the quartiles of the distributions are indicated by dashed red lines.

**FIGURE 4:**
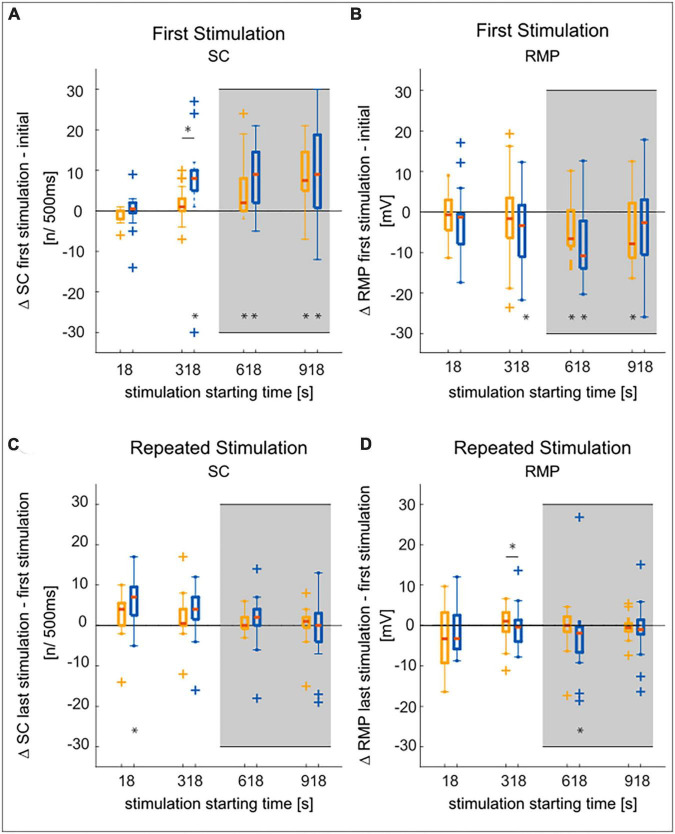
Comparison of SC and RMP changes over time in T2 and T3 cells. Statistical test decisions are based on the non-parametric Wilcoxon rank sum test. Multiple testing was Bonferroni corrected, with adjusted significance level from α < 0.05 to α’ < 0.0125, (four tests, see section “2.3.3. Statistical tests”). Significant results are given as *p < 0.0125, an asterisk below a boxplot indicates a significant deviation from zero, an asterisk above the boxplots shows significant differences between T2 and T3. T3 is shown in yellow, T2 in blue, outliers are marked by + symbols, medians are given by red lines. The gray background indicates experiments with 10 min waiting time before the stimulation with the “T-Characteristics” protocol started (see Figure 1). **(A)** The increase in spike count from the test pulse at the very beginning of the experiment to the first presentation of the “T-Characteristics” stimulation protocol increased with recording time, leading to significant deviations from zero. The increase in spike count is only significantly different between the soma locations at a stimulus starting time of 318 s. **(B)** The resting membrane potential hyperpolarized with increasing time from the initial RMP to the start of the stimulation. This effect did not differ significantly between the T3 and T2 cells. **(C)** Comparing the final to the first stimulus presentation during 5 min of repeated application of the “T-Characteristics” protocol, the tendency of increasing spike counts is mostly not significant. **(D)** The RMP did not change consistently during the repeated stimulation and was mostly neither significantly different from zero nor between cell types.

The neuronal response features (listed below) of the T cell double recordings were quantified based on the 1.5 nA current pulses in the test protocol and the “T-Characteristics” protocol, respectively. The response feature values determined in the test trial will be labeled as “Initial” in Figure 2. The prototype spike in Figure 3 is based on the second spike elicited by the test pulse. The change from the “Initial” to the first trial in the “T-Characteristics” stimulus protocol will be called “First Stimulation” in Figures 4A, B. Changes in response features spike count (ΔSC) and resting membrane potential (ΔRMP) between the last and the first trial during the repeated application of the “T-Characteristics” stimulus protocol are given as differences and will be referred to as “Repeated Stimulation” in Figures 4C, D.

#### 2.3.1. Response to somatic current injection

Figure 2 analyzes the initial responses to the first current stimulation after insertion of the electrodes into the two T cells in the double recordings. Since electrode clogging or breaking of the micropipette tip can lead to errors in the input resistance measurement, only recordings with a stable electrode resistance before, during, and after the measurement were considered in Figure 2, which reduced the number of recordings from 82 each to 61 T2 cells and 68 T3 cells for this figure. The following response features were analyzed:

• **Spike Count (SC)** is defined as the total number of spikes recorded in the soma during the 500 ms between the stimulus onset and offset for the stimulated T cell. For Figure 2, we considered the responses to the +1.5 nA pulse in the test protocol, and for Figure 4 the +1.5 nA pulse in the stimulation protocol. Spike detection was accomplished using a custom-developed MATLAB software based on the *findpeaks*-function. T cell spikes were detected with the following parameters: minimum spike distance [5.1 ms], maximum duration (at half of the prominence) [10.5 ms], and minimum spike prominence [15 mV].
• **Latency (LAT)** [ms] was defined as the time between the onset of the 1.5 nA current pulse in the test protocol or in the “T-Characteristics” protocol and the peak of the respective first spike, as determined by the *findpeaks*-function.
• **Rebound (RB)** Spike Count was defined as the number of spikes elicited by a neuron in the 1000 ms time window immediately after the −1.5 nA current pulse in the initial test protocol.
• **Input Resistance (IR)** [MΩ] was calculated from the difference between the average resting membrane potential (V_*unstim*_) and the response to a 500 ms long hyperpolarizing current pulse of *I*_*stim*_ = -1.5 nA (V_*stim*_) in the test protocol, by applying Ohm’s law:

(1) $$R\,\left[M\,\Omega \right]=\frac{V_{s\,t\,i\,m}\,\left[m\,V\right]-V_{u\,n\,s\,t\,i\,m}\,\left[m\,V\right]}{I_{S\,t\,i\,m}\,\left[n\,A\right]}$$

• **Resting Membrane Potential (RMP)** [mV] of each trial was computed as the median membrane potential in the 1000 ms before the onset of the +1.5 nA current pulse.
• **Absolute Spike Amplitude (AMP)** [mV] was defined as the difference between the maximum (spike peak) and minimum (most negative value of the afterhyperpolarization) membrane potential value within a range of 151 datapoints (from 7.5 ms before to 7.5 ms after) centered to the peak of a spike found by the *findpeaks*-function. For Figure 3, only the second spike elicited by the test pulse at the beginning of the recording was considered, since spike amplitudes change slightly over stimulation time. Cells that did not elicit at least two spikes were excluded from this analysis, reducing the sample to 54 T2 and 59 T3 cells.

#### 2.3.2. Postsynaptic response

In half of our T-T double recordings (42 recordings consisting of 23 ipsi- and 19 contralateral cell pairs), we applied 10 min of waiting time between the test stimulus and the “T-Characteristics” stimulus (see Figure 1B, gray area, and detailed description in section “2.2. Experimental design of the intracellular double recordings”) to investigate spontaneous network input. Despite the absence of electrical stimulation during the waiting time, spontaneous postsynaptic potentials (IPSPs and EPSPs) and occasional spikes (Figure 1C) appeared in many of the recorded cells. The spontaneous IPSPs during the final 5 min of the waiting time were compared to the postsynaptic responses elicited by the current injection into the other (presynaptic) T cell (10 trials of the 30 s long “T-Characteristics” protocol). Because the main aspect of our study was to record spikes to compare the excitability with previous studies (Meiser et al., 2019; Scherer et al., 2022), we did not measure postsynaptic currents but performed all recordings in current clamp.

Despite the preprocessing steps described in section “2.3. Data analysis of the intracellular double recordings,” some baseline drifts remained, which impaired the analysis of graded synaptic potentials. The baseline was adjusted with the function “msbackadj” from the MATLAB bioinformatics toolbox with the parameter values “WindowSize” = 10000, “StepSize” = 10000, “RegressionMethod” = “spline” (cubic interpolation); “EstimationMethod” = “quantile” (with default quantile value 10%), “SmoothMethod” = “rlowess” (robust linear fit). The baseline was adjusted separately for the 5 min of waiting period and for the 5 min while the presynaptic cell was stimulated. The adjusted baseline fluctuated around 0 mV, but the mean could deviate from 0 mV when deviations from the resting membrane potential were not symmetric.

For each recorded cell, we calculated the mean and the standard deviation of the adjusted recording trace during the final 5 min of waiting time. We defined the mean plus two times the standard deviation as the threshold for the presence of spontaneous EPSPs, and the mean minus two standard deviations for IPSPs. The same threshold was applied to the deviations of the postsynaptic responses from the mean membrane potential during the 5 min while the other T cell was stimulated with the “T-Characteristics” protocol.

**FIGURE 5:**
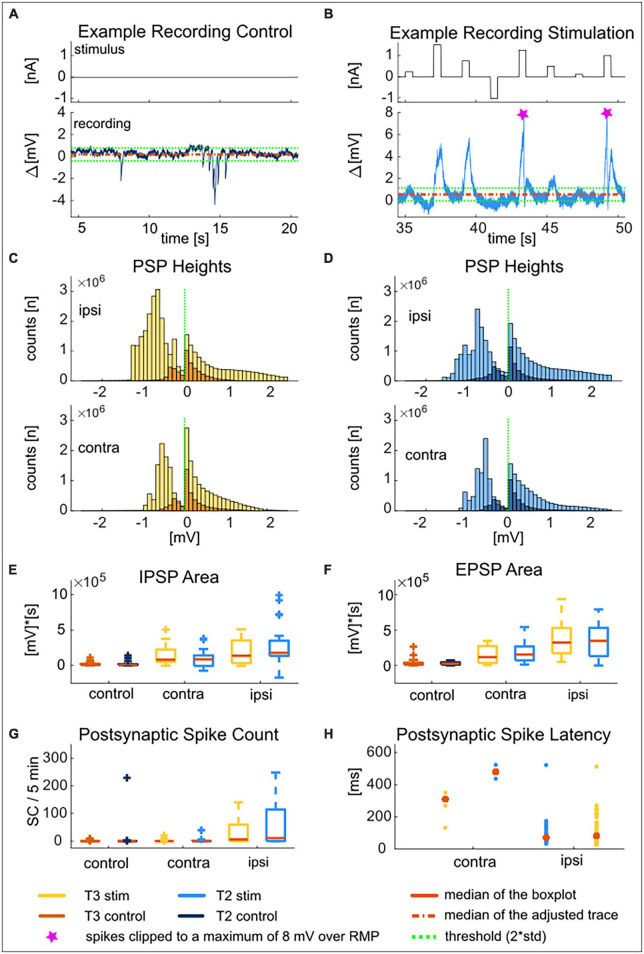
T2 and T3 cells receive spontaneous network input and react to presynaptic T cell stimulation with postsynaptic potentials and spikes. Example recording of a T2 cell during 15 s of control **(A)** and 15 s of presynaptic T3 stimulation **(B)** to show the typical response patterns. The mean of the adjusted membrane potential is shown as a dashed red line. The dashed green lines are the thresholds for EPSPs (mean + 2std) and for IPSPs (mean–2std). The two spikes that occurred during the postsynaptic response in this example trace were clipped at 8 mV over resting membrane potential (pink markers). Distribution of heights of inhibitory (left of the dashed green line) and excitatory (right of the dashed green line) postsynaptic potentials for T3 cells **(C)** and T2 cells **(D)** in ipsilateral and contralateral recording configuration triggered by the “T-Characteristics” protocol. Heights are calculated as the difference between the membrane potential and the threshold [indicated by the dashed green lines in **(A,B)** and marked by the dashed green line at 0 mV in the distributions]. Dark colors (control) show distributions of spontaneous PSP heights (5 min without stimulation), and bright colors distributions of postsynaptic response heights triggered by the presynaptically injected “T-Characteristics” protocol (5 min of repeated stimulation, “stim”). Upper panels show responses in ipsilateral, and lower panels in contralateral recording configuration. Area of inhibitory **(E)** and excitatory **(F)** postsynaptic potentials during control (*n* = 42) and during contra (*n* = 19) and ipsilateral (*n* = 23) presynaptic stimulation for soma locations T3 (yellow and orange) and T2 (light and dark blue). **(G)** Number of spikes in 5 min during spontaneous network input (control) vs. presynaptic stimulation of the contra- or ipsilateral T3 (blue) or T2 (yellow) cell. **(H)** Initial latency for the postsynaptically generated spikes of T3 (yellow) and T2 (blue) cells within 700 ms after the onset of presynaptic current stimulation with +1.5 nA of the synchronously recorded T cell on the contra- (left) or ipsilateral (right) side of the ganglion. Latencies are shown as individual data points due to the low number of spikes triggered by contralateral stimulation. The statistical test decisions corresponding to **(E–G)** are given in Supplementary Table 2.

• **PSP height** [mV]. The height of EPSPs and IPSPs were calculated as the difference between the membrane potential value and the threshold for each time point while the threshold was crossed (Figures 5C, D). All time points with threshold crossings of all cells were pooled in the histograms. The green line at value 0 mV in the histogram refers to the upper threshold for EPSPs and the lower threshold for IPSPs. PSP heights were calculated based on the threshold determined for the spontaneous network input (two standard deviations around the mean). The same threshold was applied to the postsynaptic responses elicited by the stimulation of the other T cell.

• **PSP area** [mV × s]. The total EPSP area (Figure 5F) was calculated as the temporal sum of all EPSP heights over the detection threshold and normalized to seconds for each of the cells. The IPSP area was calculated, respectively, however, as absolute value (Figure 5E). The boxplots show the distributions of the total areas obtained for all cells in the respective group. PSP areas were calculated with the same threshold for the spontaneous network input and for the input from the stimulated other T cell.
• **Postsynaptic Spike Count**. The total number of spikes during the 5 min of the control and the stimulation periods was counted for each cell by using the spike detection method described above (see section “2.3.1. Response to somatic current injection,” SC).
• **Postsynaptic Spike Latency** [ms]. If spikes were detected in the postsynaptic cell within 800 ms after the onset of the presynaptic stimulation with +1.5 nA, the time difference between the time of the first postsynaptic spike and the presynaptic stimulus onset was determined as postsynaptic spike latency. The more common spike-triggered postsynaptic spike latency is not applicable to our data set, because it is not possible to determine a 1:1 relationship between one pre- and the corresponding postsynaptic spike. Most stimuli trigger several spikes in the stimulated presynaptic cell. Their interspike intervals are much shorter than the synaptic latency, making it impossible to determine which of the presynaptic spikes triggered the postsynaptic response. Since the number of postsynaptic spikes triggered in the contralateral configuration was too low for a statistical analysis, Figure 5H shows the latencies of all cells with 10 stimulus presentations each.

#### 2.3.3. Statistical tests

**FIGURE 6:**
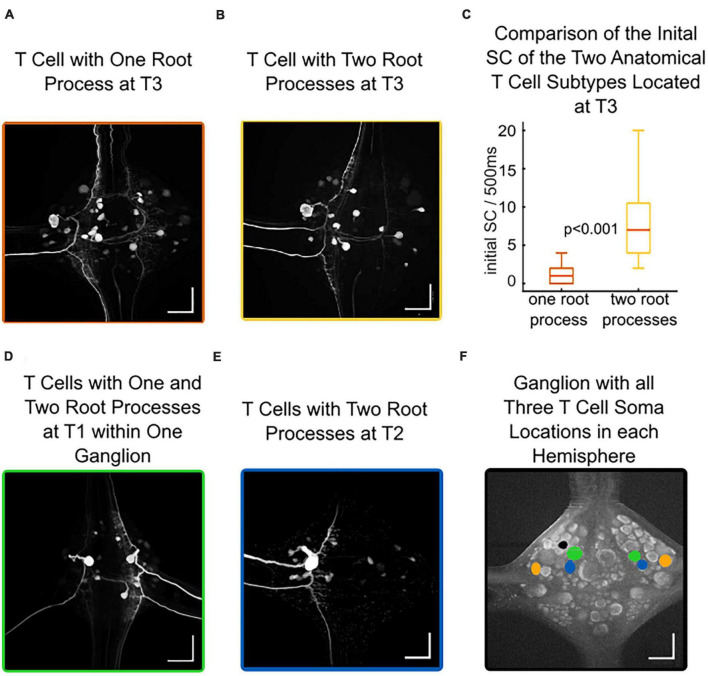
Different anatomies of T cells at the same location correlate with different spike counts. Intracellular Neurobiotin injections into T cells revealed their morphology and the somata of electrically coupled neurons. All scale bars refer to 100 μm. **(A)** An example of a T cell with soma location at T3 and one root process, potentially innervating the dorsal receptive field. **(B)** An example of a T cell with soma location at T3 and two root processes, potentially innervating the lateral or ventral receptive field. **(C)** Initial spike count of T3 cells at soma location T3 for 21 cells with one branch (dorsal receptive field), and 25 cells with two branches (lateral or ventral receptive field) differ significantly (non-parametric Wilcoxon rank sum test). **(D)** Both T cells at soma location T1 in one ganglion, on one side with one branch (putatively dorsal receptive field), on the other side with two branches (putatively lateral or ventral receptive field). **(E)** An example of a T cell with soma location at T2 and two root processes, potentially innervating the lateral or ventral receptive field **(F)** Autofluorescence picture of a ganglion, indicating the location of all three T cell soma locations (green: T1, blue: T2, orange: T3) in each hemisphere. The black dot is an artifact from the Alexa-filled interneuron (see section “2.4. Anatomical studies”).

Direct comparisons between the two distributions obtained for T2 vs. T3 cells (Figures 2–5), and for T3 cells with one vs. two root branches (Figure 6) were performed with the non-parametric Wilcoxon rank sum test. For paired tests, deviations from zero were tested with the non-parametric Wilcoxon signed-rank test (Figures 4, 5). All statistical tests were applied to the median values obtained for each of the cells in the respective sample, the sample sizes are listed in Supplementary Table 2. For comparisons in which datasets were used for multiple testing (Figures 4, 5), we used the Bonferroni’s method for correcting the significance level of α = 0.05 to α’ = α/4 = 0.0125 for Figure 4 [For each cell, the response to the first stimulus presentation is used for the tests (1) difference from the initial response, (2) difference from the response to the final stimulus presentation, (3) comparison of differences from the initial response between T2 and T3, and (4) comparison of differences from final response between T2 and T3]. In Figure 5, the corrected significance level is α’ = α/3 = 0.0167 [e.g., EPSP T2 ipsilateral stimulated is tested vs. (1) spontaneous ESPSs in the same recordings, (2) ipsilateral EPSPs in T3, (3) contralateral EPSPs in T2]. All results of statistical tests in this study are summarized in Supplementary Table 2, where all *p*-values are rounded to the fourth decimal place.

### 2.4. Anatomical studies

For anatomical reconstructions, T3 cells in isolated ganglia were filled with 2% Neurobiotin in potassium chloride (NB, Vector Labs, Peterborough, UK) using sharp microelectrodes (∼40–90 MΩ). Cells were iontophoretically injected with positive current pulses (3 nA, 1 Hz, stimulus 750 ms, break 250 ms) for up to 40 and 15 min inside the cell without stimulation.

After injection, NB-samples rested for 45 min in a dark box before further processing and being fixed in PFA-Fix [0.1 M PB pH 7.4; 4% formaldehyde (Sigma, Munich, BY, Germany)] for 1.5 h. After 6 × 10 min washout in 0.1 M PBS (pH 7.4), the NB-samples were incubated in 1:500 Streptavidin conj. Cy3 (Vector Labs, Peterborough, UK)/PBS/0.3% Triton-X100 for 18 h overnight at 4°C. The next day, they were washed 6 × 10 min in 0.1 M PBS (pH 7.4) and embedded with Vectashield (Vector Laboratories, Burlingame, CA, USA) on a microscope slide for high resolution microscopy.

The NB-samples were scanned with a Leica TCS SP 2 Confocal Microscope (Leica, Nußloch, BW, Germany) using an APO 20 × air objective. We obtained confocal stacks of different heights depending on the anatomical structure of the neuron and the ganglion. Channel overlay and adjustment of contrast and brightness were performed with the software Fiji (Schindelin et al., 2012; Bankhead, 2014).

For the overview picture of a ganglion in Figure 6F, we injected an interneuron with Alexa 647 by applying negative current steps. To visualize the position of the cell bodies on the ventral side of the ganglion, we boosted the autofluorescence artifacts by manually restricting the image intensity values to a range from 1 to 10. The black circle on the picture is the soma of the stained cell, which had an intensity value outside of this range.

We generated 3D anatomical structures of in total 17 T3 cells (8 with two root processes, 9 with one root process) as a basis for our multi-compartment modeling. We performed background subtraction, applied a median filter of 3 × 3 pixels, and a 3D mean filter, followed by triangular thresholding to prepare confocal scans, as recommended by the developers of Fiji (Bankhead, 2014) for automatic processing with the Fiji Plugin SNT (Arshadi et al., 2021). For fitting the traced anatomies, the radii were restricted to 2× of the largest radius in the traced structure (Arshadi et al., 2021). The resulting anatomical structure was saved as an swc-file.

### 2.5. Multi-compartment model

To reproduce electrical properties and response characteristics of T3 cells, we implemented a conductance based multi-compartmental model in Brian 2, version 2.5.1 (Stimberg et al., 2019) using Phyton 3.8.15. All parameter values are listed in Table 1. The anatomical structure of connected compartments was reconstructed automatically by importing the previously generated swc-files using the built-in *morphology.from_file()* function of Brian 2. Neighboring compartments were connected with an axial resistance *R*_*i*_. Their number varied across anatomies from 1072 to 2053.

**TABLE 1:** Simulation parameters.

Parameter	Standard value	Min; max; step size for sweeps
*R*_*i*_	5 Ω m	1 Ω⋅m; 1001 Ω⋅m; 100 Ω⋅m
*C*_*m*_	1 μF/cm^2^	
*E*_*K*_	−60 mV	−100 mV; −50 mV; 5 mV
*E*_*Na*_	10 mV	
*E*_*L*_	−45 mV	−60 mV; 10 mV; 7 mV
*g*_*Ksoma*_	2 mS/cm^2^	0 mS/cm^2^; 40 mS/cm^2^; 4 mS/cm^2^
*g*_*Kprocess*_	6 mS/cm^2^	1 mS/cm^2^; 41 mS/cm^2^; 4 mS/cm^2^
*g*_*Na*_	2000 mS/cm^2^	
*g*_*Msoma*_	0 mS/cm^2^	
*g*_*Mprocess*_	4 mS/cm^2^	
*g*_*L*_	0.044 mS/cm^2^	0.01 mS/cm^2^; 0.11 mS/cm^2^; 0.1 mS/cm^2^
n_1;_ n_2_	18 mV; 7 mV	
m_1;_ m_2_	12 mV; 7 mV	
h_1;_ h_2_	42 mV; −7 mV	
n_3_, m_3_, h_3_	0.1	
z_1;_ z_2;_ z_3_	40 mV; 4 mV; 80	
T_n;_ T_m;_ T_h;_ T_z_	2 ms; 0.6 ms; 3 ms; 80 ms	

T cell model parameters and value ranges for parameters investigated in the parameter sweeps.

The dynamics of the membrane voltage in each compartment was calculated as follows:


(2) $$C_{m}\,\frac{d\,V}{d\,t}=I_{N}+I_{K}+I_{M}+I_{L}$$

Where *C*_*m*_ is the membrane capacitance, *V* is the membrane voltage at time point *t* and *I*_*X*_ is the transmembrane current for each ion channel or current type. We used three voltage gated ion channels and a leak current to simulate transmembrane currents. The fast transient sodium channel (Johansen, 1991) was formulated as:


(3) $$I_{N\,a}=g_{N\,a}\cdot m^{4}\cdot h\cdot \left(E_{N\,a}-V\right)$$

Where *g*_*Na*_ is the conductance density, *m* and *h* are gating variables and *E*_*Na*_ is the reversal potential of Na^+^. The sodium currents are modeled with identical parameters in all compartments.

We used a potassium channel with two activation gates that generates a delayed rectifying K^+^ current *I*_*K*_ (Johansen, 1991) and was calculated analogous to the *I*_*Na*_ current, using the following formula:


(4) $$I_{K}=g_{K}\cdot n^{2}\cdot \left(E_{K}-V\right)$$

With potassium conductance density *g_*K*_*, reversal potential *E*_*K*_, and gating variable *n*. The values of *g*_*K*_ differ between *g_*Ksoma*_* = *2 mS/cm^2^* in the soma and *g_*Kprocess*_* = *6 mS/cm^2^* in all other compartments (see Table 1).

We added a second M-type slow potassium channel to all compartments, except for the soma. Its slower kinetics cause cessation of spiking during current injection (Benda and Herz, 2003). The M-type current was calculated with conductance density *g*_*M*_, gating variable *z* and the same reversal potential *E*_*K*_ as follows:


(5) $$I_{M}=g_{M}\cdot z^{2}\cdot \left(E_{K}-V\right)$$

In addition to the voltage dependent ion channels, a leak current was simulated with conductance density *g*_*L*_ and reversal potential *E*_*L*_ and identical values for all compartments:


(6) $$I_{L}=g_{L}\cdot \left(E_{L}-V\right)$$

Kinetic equations for each voltage-dependent ion channel were defined by:


(7) $$\frac{d\,x\,\left(t\right)}{d\,t}=\frac{x_{\infty }\,\left(V\right)-x}{\tau_{x}\,\left(V\right)}$$

Where *x* is either *m*, *h*, *n*, or *z*, *x*_∞_ corresponds to the respective steady state value and *τ_*x*_* is the respective time constant. For each of these gating variables, the steady state function was calculated as follows:


(8) $$x_{\infty }\,\left(V\right)=\frac{1}{\left(1+\exp\left(-\frac{V+x_{1}}{x_{2}}\right)\right)}$$

Time constants for each gating variable were calculated as follows:


(9) $$\tau_{x}\,\left(V\right)=T_{\text{x}}\cdot \left(\frac{2}{\exp\left(-\frac{V+x_{1}}{x_{2}}\right)+\exp\left(\frac{V+x_{1}}{x_{2}}\right)}+x_{3}\right)$$

The parameter values for conductance densities *g*_*x*_, reversal potentials *E*_*x*_, steady state function parameters *x_1–3_* and the time constant variables *T*_*x*_ are listed in Table 1.

Parameters were selected to fit the experimentally measured electrical properties and response features of a T3 cell including the RMP, IR, SC, latency, and the absolute spike amplitude. For each feature we applied the criterion of being within the range of observed values for T3 cell measurements. The fitting process was performed iteratively for each of the T3 cell anatomies by adjusting the parameter values listed in Table 1. First, the parameters were fitted for one example of each of the two anatomical subtypes to the medians of the experimentally determined response features. Then, the other anatomies were tested with the same parameter set and the parameters were adjusted until all anatomies yielded response features within the ranges of the corresponding measured distributions.

To investigate the robustness of the parameter set, we additionally performed parameter sweeps with one variable being varied at a time around its standard value. During the fitting process, the impact of the input resistance on other response features was revealed. Therefore, we decided to vary all parameters that have a crucial impact on the input resistance (Table 1, right column). Minimal and maximal parameter values for parameter sweeps were determined by in- and decreasing the parameter value until the model failed in at least one of the three criteria: (1) A stable resting potential in the experimentally observed range during the entire un-stimulated simulation period. (2) Hyperpolarization during negative current injection. (3) Action potential generation in response to positive current injection.

## 3. Results

The current study aims to reveal the possible reasons for the previously observed high variability in leech T cell responses. We compare the responses of T cells that have their soma at T3 location (“T3 cells”) to the responses of T cells with the soma at T2 location (“T2 cells”). In intracellular double recordings, the membrane potential of the mechanosensory T3 and T2 cells in contralateral (Figure 1A) and ipsilateral (Figure 1B) configuration was measured, with three stimulus protocols applied consecutively (for details see section 2.2, and Figure 1C). After an initial test stimulation, the main stimulus protocol (“T-Characteristics”) was applied first to the left and then to the right electrode to record the synaptic interaction of the two T cells. The experimental design and an example ipsilateral double recording of a T2 cell (blue) and a T3 cell (yellow) are presented in Figure 1C.

### 3.1. T cells at soma location T2 are more excitable, respond faster, and generate larger spike amplitudes than at location T3

Figure 2 shows the distributions of the initial response features spike count (SC), first spike latency (LAT), rebound spike count (RB), cell input resistance (IR), and resting membrane potential (RMP) triggered by the test protocol in 68 cells with their soma at location T3 (yellow) and 61 cells at location T2 (blue). Supplementary Table 2 lists the sample sizes and *p*-values of all statistical test decisions.

The initial SC in response to the 500 ms long current pulse of +1.5 nA was highly variable with a range from 0 to 20 spikes for T3 cells (Figure 2A), consistent with the results by Scherer et al. (2022). T2 cells also responded with a broad distribution of 1 to 30 initial spikes. The initial spike counts of T2 cells were found to be significantly higher than at location T3 (Figure 2A, median T2 = 11, median T3 = 4). Furthermore, T2 cells responded with a significantly shorter latency to the positive current pulse in the test protocol than T3 cells (Figure 2B). The latency distribution covers the range from 5 to 20 ms and is skewed for cells recorded at both soma locations. The median response latency was significantly shorter (Supplementary Table 2) in T2 (6.9 ms) than in T3 cells (9.0 ms).

Also, the number of rebound spikes after a −2 nA current pulse was significantly higher for T2 cells (median 3 RB spikes) in comparison to T3 cells (median 2 RB spikes, Figure 2C), probably at least partly due to a higher percentage of cells without any rebound spikes at soma location T3 than at T2.

In contrast to the spike features, the passive properties input resistance and resting membrane potential did not differ significantly between the T cell populations (see Supplementary Table 2). T2 cells had a slight tendency toward a higher median initial input resistance than T3 cells (median 31.6 vs. 26.7 MΩ), with broad distributions (ranging from less than 10 MΩ to more than 70 MΩ) at both soma locations (Figure 2D). The initial RMP ranged approximately from −65 to −35 mV for T cells at both soma locations (Figure 2E).

Figure 3A compares the average spike shapes of T2 and T3 cells. T2 spikes (blue) reached on average a higher amplitude, mainly due to a more prominent afterhyperpolarization than T3 spikes (yellow). With an ∼10 mV higher median spike amplitude in T2, the difference in absolute spike amplitude is highly significant (Figure 3B). In contrast, the spike shape and width showed no obvious differences between T2 and T3 cells (Figure 3A).

In summary, the initial response features of T cells at soma locations T3 and T2 were found to be highly variable within each soma location. T cells at soma location T2 are significantly more excitable, react faster and have a higher absolute spike amplitude than at location T3. In contrast to the spike properties, the resting membrane potential and the input resistance are not significantly different between both T cell locations. In the following sections, we will probe three possible reasons for these findings.

### 3.2. The time-dependent changes in response features do not differ between T2 and T3

The time-dependent increases in excitability of T cells reported by Meiser et al. (2019) and Scherer et al. (2022) could contribute to the high variability of spike counts between cells. Here, we investigate if these time effects could be a reason for the significant differences in the initial responses of T cells at soma locations T2 vs. T3 by comparing the responses of the same stimulation applied at different starting times (see Figure 1C and see section “2.2. Experimental design of the intracellular double recordings”).

In accordance with the findings of Scherer et al. (2022) we found for both soma locations that more spikes were generated after a later starting time of the current stimulation with the “T-Characteristics” protocol (Figure 4A). Nevertheless, only when the stimulation started immediately after the test pulse (at 18 s, Figure 4C) the T2 cells increased their excitability significantly during the repeated presentation of our stimulation protocol (see Supplementary Table 2 for all *p*-values). After a longer waiting time, the SC increase during these 5 min of stimulation was not significant, probably due to the massive increase in SC that already happened during the waiting time before the stimulation (compare Figure 4A).

Furthermore, the RMP measured at the beginning of the stimulation protocol was more hyperpolarized for later starting times of the stimulation with the “T-Characteristics” protocol (Figure 4B). However, the change in RMP measured between start and end of the 5 min long stimulation (Figure 4D) was not significant, except for T2, when the stimulation started 10 min (at 618 s) after the test pulse. The time-dependent changes in RMP were mostly not significantly different between T2 and T3 cells (Supplementary Table 2).

In summary, we found that the spike count of T cells at both soma locations increases with time, with the most prominent change at the beginning of the recording time. T2 cells seem to have the tendency of a higher increase in SC compared to T3 cells, but these differences are not statistically significant (Supplementary Table 2). Hence, we conclude that the time-dependent changes in excitability, which are so characteristic for T cells (Meiser et al., 2019; Scherer et al., 2022), can contribute to the variability between individual cells, but are not the major reason for the systematic differences between T cells at the two locations.

### 3.3. T2 and T3 cells do not differ in their spontaneous input from the network and reaction to presynaptic T cell stimulation

It is known for decades that T cells, despite being primary mechanoreceptors, receive synaptic inputs from the network, including spontaneous IPSPs, and that all 6 T cells in each ganglion are mutually coupled (Baylor and Nicholls, 1969b). In this part of our study, we perform simultaneous T2–T3 double recordings to investigate if the systematic differences between T cells at soma locations T2 and T3 could stem from distinct network input.

In the first step, the spontaneous synaptic input from the network to both recorded T cells was analyzed in the recording without stimulation. We frequently observed spontaneously occurring large IPSPs. EPSPs were more numerous, but often smaller than IPSPs (Figure 5A and dark histograms in Figures 5C, D). The distributions of PSP heights observed in T3 and T2 cells strongly resembled each other (Figures 5C, D). The total areas of the spontaneous IPSPs and EPSPs did not differ significantly between our samples of T cells at both soma locations (control in Figures 5E, F, and Supplementary Table 2).

In 42 T2-T3 double recordings, only five of the T2 and three of the T3 cells spiked in response to the spontaneous network activity. While cells at both locations generated 0 spikes in median (Q_1_ = 0, Q_3_ = 0.5) during the 5 min of control, in one individual cell the spontaneous activity was as large as 200 spikes in 5 min (Figure 5G). Like for the EPSP and IPSP areas, the number of spikes elicited by spontaneous input from the unstimulated network was not significantly different between the two T cell locations (Figure 5G and Supplementary Table 2).

In the simultaneous double recordings, we compared the spontaneous synaptic activity of each T cell to its postsynaptic responses that were triggered by current injection into the presynaptic T cell. Qualitatively, many more and larger EPSPs were observed during the periods of positive current injection into the presynaptic cell (Figure 5B). These EPSP often triggered postsynaptic spikes (clipped to 8 mV over mean resting potential in Figure 5B). When applying the threshold that was determined for the control recording (mean resting potential ± 2 × standard deviation of the control) to the postsynaptic response of the same neuron, larger EPSP heights became evident in the distributions (compare dark spontaneous and light stimulus-induced distributions in Figures 5C, D, positive values on *x*-axis, heights clipped to + 2.5 mV over threshold). The lower threshold was crossed more often and with larger heights, (Figures 5C, D, negative values on *x*-axis). These large hyperpolarization events mainly came from action potential afterhyperpolarization rather than individual IPSPs. Qualitatively the same effects were observed for double recordings in ipsi- and in contralateral configuration (Figures 5C, D, upper vs. lower panels). However, the increase in PSP heights appeared to be more pronounced for synaptic inputs from the ipsilateral side of the ganglion.

Quantitative comparison of the total IPSP and EPSP areas per cell confirmed that presynaptic T cell stimulation with the “T-Characteristics” protocol led to a significantly higher PSP area of the postsynaptic responses in coupled T cells on both sides of the ganglion (Figures 5E, F, see *p*-values of Bonferroni corrected non-parametric Wilcoxon rank sum test in Supplementary Table 2). The PSP area did not differ significantly between the ipsi- vs. contralateral stimulation, except for EPSPs in T3 cells. The direct comparison between T2 and T3 cells did not yield a significant difference in the magnitude of the postsynaptic response in any of the experimental configurations.

The numbers of postsynaptic spikes did not differ significantly between the soma locations, neither in ipsi- nor in contralateral configuration. In contrast, both T2 and T3 cells responded with significantly more postsynaptic spikes to inputs coming from ipsilateral than from contralateral T cells or in the unstimulated condition (Figure 5G). The few postsynaptic spikes occurred with a very long latency after the onset of the presynaptic +1.5 nA stimulus. The measured latency was considerably shorter for ipsilateral inputs (medians: 70.25 ms in T2, 80.9 ms in T3, no significant difference between soma locations) than for the few contralateral inputs that triggered a postsynaptic spike after several hundred of milliseconds (Figure 5H).

In summary, there is no indication that the T cells at the two soma locations T2 and T3 receive a different amount of spontaneous input from the unstimulated network, and they respond to synaptic input from polysynaptically coupled T cells in the same way. We conclude that synaptic interactions might contribute to the response variability, but differential network inputs are probably not the major cause for the systematic differences in excitability of T cells at soma locations T2 vs. T3.

### 3.4. Different anatomies of T cells at the same location correlate with different spike counts

Intracellular neurobiotin injections into T cells revealed their morphology and the soma locations of other electrically coupled neurons in the same ganglion. Figure 6A shows an example of a T cell with soma location T3 and one root process stretching via the dorsal branch of the posterior root putatively onto the dorsal receptive field in the skin. Figure 6B also shows a T3 cell, but with two root processes that putatively innervate the ventral or lateral receptive field in the skin (Nicholls and Baylor, 1968). This T3 cell with two branches shares a similar anatomical structure with the T2 cells (Figure 6E). Overall, we found one-branched T cells in 21 out of 46 stained cells with soma location T3, and in 4 out of 11 at soma location T1. In contrast, all (18 out of 18) stained T cells at soma location T2 had two root processes. Additionally, T cells at the same soma location can have different anatomies in both hemispheres of a ganglion. Figure 6D shows a T1 cell (anterior to the T2 location, see Figure 6F) at the left side with one root process (probably innervating the dorsal receptive field) and another T1 cell with two root processes (probably innervating the lateral or ventral receptive field) on the right side.

When relating the physiology of 46 T3 cells to their anatomy, we discovered that T3 cells with one root process responded with a median SC of only 1 spike to our 1.5 nA test pulse. In contrast, T cells at the same T3 soma location but with two root processes had a median spike count of 7 spikes. The most active T3 cell with one root process fired 4 spikes, while the spike counts in T3 cells with two root processes reached up to 20 spikes, leading to a highly significant difference between both anatomical subtypes at the same soma location T3 (Figure 6C and Supplementary Table 2). This finding could explain the significant difference between T2 and T3 cells displayed in Figure 2A, in this larger dataset 50% of the T3 cells responded with 4 or fewer spikes to a 1.5 nA current pulse of 500 ms duration. The (presumably predominantly two-branched) T2 cells were significantly more active with a median of 11 spikes in 500 ms, and only few T2 cells had a low initial spike count.

In conclusion, our histological results show, in accordance with Nicholls and Baylor (1968), Yau (1976), and Kretzberg et al. (2016), that the three ipsilateral T cells with distinct receptive fields in the skin do not always have the same soma location in every ganglion. However, the probabilities for innervating one of the three receptive fields in the skin are not evenly distributed over the three soma locations. In our sample of 75 stained T cells, the anatomical subtype with only one root process, which presumably innervates the dorsal receptive field (Nicholls and Baylor, 1968), was found at T3 and T1 location. We did not find the one-branched T cell subtype at T2, while previous studies also reported it at this soma location (Nicholls and Baylor, 1968; Kretzberg et al., 2016). Moreover, we found a correlation between the initial spike count and the two anatomical subtypes of T3 cells. T3 cells with only one root process are less active than T3 cells with two root processes and T2 cells. Therefore, the final step in our analysis is to test if the higher excitability is caused by the anatomical feature of a second root process.

### 3.5. Individual anatomical differences between T cells cause variability in neuronal responses but cannot explain the systematic differences between the anatomical subtypes

We used the 3D anatomical structure of 17 filled T3 cells as a basis for simulations with detailed multi-compartment models. In our dataset, 9 of the T3 cells had one root process, and 8 had two root processes. The simulations were performed deterministically and with identical biophysical parameter sets that include the capacitance of the membrane, resistivity of the branching processes, and distribution of ion channel conductances, leaving the individual anatomical structures as only source of variability. Since no experimental data on channel distributions was available, we used identical Na^+^ and K^+^ conductances in all compartments, except for the soma. The models were fitted to the experimentally determined initial responses of T3 cells (Figures 2, 3), specifically the response features of resting membrane potential, input resistance, spike count, spike height, and spike latency measured in response to 500 ms of 1.5 nA current stimulation. The biophysical model parameters (Table 1) were selected to yield simulation results for these five response features within the ranges experimentally observed in T3 cells (Figures 2, 3) for all 17 neuronal anatomies.

**FIGURE 7:**
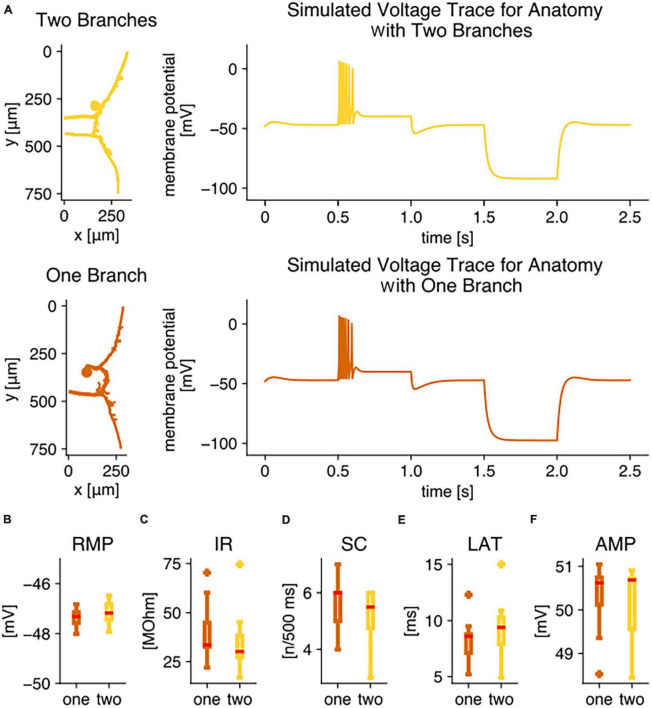
Compartmental modeling reveals that the two different anatomies do not lead to the observed physiological differences. Simulation results of compartmental models for 17 detailed T3 cell anatomies, 9 with one branch, 8 with two branches, obtained from Neurobiotin stainings. **(A)** Example anatomical structures and voltage traces in the soma calculated with the identical set of simulation parameters for the anatomies of an exemplary T3 cell with two branches (yellow) and an example T3 cell with one branch (orange). For these two examples, the response feature values were for the version with two branches (yellow) RMP = –46.9 mV, IR = 30.1 MΩ, SC = 6, LAT = 9.3 ms, AMP = 50.8 mV. For the version with one branch (yellow) the feature values were RMP = –47.1 mV, IR = 33.6 MΩ, SC = 6, LAT = 8.6 ms, AMP = 50.7 mV. Boxplots of simulation results of physiological parameters, comparing responses to +1.5 nA current injection for 500 ms of all 9 anatomies with one branch (orange) to all 8 anatomies with two branches (yellow): **(B)** resting membrane potential, **(C)** input resistance, **(D)** spike count, **(E)** response latency, **(F)** absolute spike amplitude. All simulations were performed with the identical simulation parameter set (Table 1) that was fitted to yield response features in the experimentally observed range (Figure 2).

Figure 7A shows the reconstructed anatomical structures (left) and resulting simulated responses (right) of two model examples. When applying the same test stimulation for the initial responses as in our electrophysiological recordings, both models produced biologically realistic membrane potential responses to positive and to negative current injection, replicating the experimental results (compare Figure 1C). The simulated voltage trace of the model with two root processes (yellow) closely resembled the trace of the model with one root process (orange).

The similarity between responses simulated with models of both anatomical subtypes applied to the entire set of 17 simulated T3 cells with individual anatomies. The resting membrane potential was within a 1.5 mV range for all models, independent of their anatomies (Figure 7B). In contrast, the input resistance varied strongly between models with individual anatomical structures. They covered a range from 16.8 to 74.5 MΩ, despite the fact that an identical set of biophysical model parameters was used for all of them (Figure 7C). Nevertheless, the two anatomical subtypes did not lead to a difference in the input resistance distributions (Figure 7C). With 3 to 7 spikes in 500 ms, the models produced responses typical to T3 cells (compare Figures 2A and 7D). Again, the models did not reproduce the significantly different spike counts between anatomical subtypes with one vs. two root processes (Figure 7D). Additionally, the response latency (Figure 7E) and the absolute spike amplitude (Figure 7F) were found to vary in a biologically realistic range between individual model anatomies, but no significant difference was found between both anatomical subtypes. To test the robustness of the model, we selected model parameters that have crucial effects on the input resistance and varied them around their standard values (see section “2.5. Multi-compartment model” and Table 1, right column). The major finding that the responses of both anatomical subtypes do not differ systematically was conserved for a wide range of biophysical model parameter values.

In conclusion, the detailed individual anatomical structure impacts all response features tested, except for the resting membrane potential, which depends mainly on the model’s leak reversal potential. In particular, the experimentally observed high variability in input resistances (Figure 2D) can be reproduced by models with identical biophysical parameters but with individually different anatomical structures (Figure 7C). However, the number of branches does not correlate with any of the response features. Hence, the experimentally observed systematic differences in excitability between T3 cells with one vs. two root processes cannot be explained solely by their distinct anatomies.

## 4. Discussion

The leech mechanosensory T cell is a well-known cell type that was studied for many decades (Nicholls and Baylor, 1968). The three bilateral pairs of T cells in each ganglion innervate distinct receptive fields in the skin (Blackshaw, 1981). Concerning their electrophysiological properties, however, all T cells were considered to belong to one homogeneous cell type, independent of their receptive fields or detailed anatomies (Burrell, 2017). This assumption is challenged by our present finding that the T cells at soma location T3 are significantly less excitable than at soma location T2. In response to somatic current injection, T3 cells generate fewer spikes with a longer response latency and smaller spike amplitudes than T2 cells (Figures 2, 3). This finding explains an apparent difference between two previous publications: The initial spike count in the T3 cell population recorded by Scherer et al. (2022) was significantly lower than in the data set of Meiser et al. (2019), which comprised T cell recordings from all three soma locations, including many T2 cells.

In this study, we systematically analyzed three factors of neuronal response variability that could potentially be the reason for the systematic differences between T cells at different soma locations: Time-dependent activity changes, synaptic inputs from the unstimulated and the stimulated network, and differences in neuronal anatomy. While we found that all three factors contribute to the response variability within the T cells we recorded at each of the two soma locations, we ruled out that any of the three factors by themselves can explain the systematic differences between the T cells at locations T2 vs. T3.

### 4.1. Effects of time-dependent activity changes

We found that the number of spikes in T cell responses to somatic current injection increased over recording time, in particular during the first 5–10 min of recording. This effect, as well as the hyperpolarization of the membrane potential during long recordings, was present in all T cells, without a statistically significant difference between the soma locations. These results are consistent with a previous study in which time-dependent response changes were shown to be specific to mechanosensory T and P cells. The increase in excitability did not occur in Retzius cells, and could not be explained by any potential experimental confounders (Scherer et al., 2022). In the present study, experimental conditions were identical for the synchronously recorded T cells at both soma locations. Hence, factors like temperature, which globally influence neuronal responses, should have had an equal impact on both cells in the same recording. Local factors like variable electrode resistances also did not have a systematic effect on T2 vs. T3 cells, because they varied in the same range for all recordings. Moreover, systematic bias was prevented by impaling half of the cells at each soma location with the left and the other half with the right electrode.

Our findings can explain the differences in the initial spike counts observed by Meiser et al. (2019) compared to Scherer et al. (2022) by the different percentages of T2 vs. T3 cells in their samples. However, despite our precautions to prevent systematic errors, the results on time-dependent response changes differ quantitatively between our results in this study and two previous publications (Meiser et al., 2019; Scherer et al., 2022). The higher time-dependent increase in excitability reported by Meiser et al. (2019) seems not to be due to differences between T2 and T3 cells. Since T cells at both soma locations increased their excitability in the same way during our recording time, it seems not to be plausible that previous distinct time-dependent changes in excitability could have caused the observed systematic differences in the initial responses of T2 vs. T3 cells.

### 4.2. Effects of synaptic inputs

The fact that T cells, despite being primary mechanoreceptors, receive excitatory and inhibitory synaptic input from the network (Baylor and Nicholls, 1969a; Burgin and Szczupak, 2003) including mutual connections with the other T cells in the ganglion (Baylor and Nicholls, 1969b; Gu et al., 1989) was confirmed by our study. It should be kept in mind that even postsynaptic potentials (PSPs), that do not trigger a spike that reaches the soma, still have important biological functions. PSPs interact in dendritic integration (Stuart and Spruston, 2015) and, depending on the level of electrical compartmentalization, thereby impact the electrical activity in smaller or larger parts of the neuron (Francioni and Harnett, 2022). Thereby, PSPs potentially also contribute to location-dependent synaptic plasticity (Weber et al., 2016). Modeling studies suggested that subthreshold signals are transmitted in complex networks of spiking neurons (Torres et al., 2015). It is plausible that subthreshold signals can impact the network without ever reaching spike threshold, because leech T cells share electrical connections with several other neurons (Segura et al., 2020). Even in an isolated ganglion without network stimulation, PSPs are visible in the T cell membrane potential recordings. Periods of multiple spontaneous IPSPs with heights of up to 4 mV (see examples in Figures 1, 5) resembled the reafferent inhibition that T cells receive from the motor system during fictive crawling (Alonso et al., 2020). These large inhibitory events were reported to occur synchronously in ipsilateral pairs of T cells (Baylor and Nicholls, 1969b). We observed them more frequently in the unstimulated ganglion, but they also sometimes coincided with the synaptic potentials elicited by presynaptic current injection into one of the other T cells (see Figure 1C, orange trace in Experiment 1). In total, the amount of spontaneous input differed greatly between preparations, but not consistently between T2 and T3 cells.

T cells on the same side of the ganglion are mutually connected via strongly rectifying electrical synapses (Baylor and Nicholls, 1969b; Gu et al., 1989). In our double recordings, presynaptic spikes evoked large EPSPs. The timing of these EPSPs matched with presynaptic positive current injection and they often triggered spikes, while negative current only sometimes caused shallow IPSPs in the unstimulated cell (Figure 5). Adjacent T cells share several widely distributed connection points of their membranes, with approximately 200 contacts in ventral and lateral T cells. In contrast, only 100 contacts were found for the one dorsal T cell that was included into the study by Gu et al. (1989). Since the electrical connection is mutual, we expect that in an ipsilateral cell pair both cells should receive fewer input from the recorded partner if the pair contains the dorsal T cell than in a pair consisting of the dorsal and the lateral T cell. Since all our recordings were performed on T2–T3 cell pairs with a probability of approximately 50% for containing a dorsal T cell, this morphological difference could probably not lead to differences between the PSPs observed in T2 vs. T3 cells.

The observed postsynaptic responses were not as reliable as one might naively expect for electrically coupled neurons. In our recordings, we frequently saw postsynaptic responses that were precisely timed to the first presentations of the presynaptic stimulation protocol but became more variable over time. This anecdotical observation might be an indication for gap junction plasticity, as it was demonstrated electrophysiologically for the electrical coupling of leech Retzius cells (Welzel and Schuster, 2018). Gap junction permeable tracer injection into dorsal T cells revealed a highly variable number of electrically coupled neurons. This number depends on extracellular ATP, suggesting modulation of network interactions (Segura et al., 2020). Hence, the modulation of gap junctions between pairs of T cells and other cell types probably contributes to the response variability between individual cells and maybe also over recording time. Additionally, ipsilateral T cell pairs are also indirectly coupled via interneurons, inducing delayed EPSPs and IPSPs superimposing the direct large electrical response (Baylor and Nicholls, 1969b). However, all these observations apply to T cells at soma location T2 and T3 and there is no evidence for systematic differences between them.

Such a strong coupling between T cells might at first glance be counterintuitive, because this could impair the ability of these mechanoreceptors to signal touch stimuli in their distinct receptive field to the network. However, since T cells encode the stimulus location by spike timing rather than spike count (Pirschel and Kretzberg, 2016), the strongly delayed synaptically induced spikes might not hamper sensory coding. On the contrary, the increase in spike count by mutual activation might be beneficial, since the excitation of one single T cell is not capable of eliciting strong network activity (Fathiazar et al., 2018) or a behavioral response (Kristan, 1982). In combination, multiple T cells might be able to activate a preparatory network preceding multiple behaviors, as proposed by Frady et al. (2016).

In contrast to the ipsilateral T2–T3 cell pairs, T cells are not electrically coupled across the midline of the ganglion, but still receive polysynaptic input via chemical synapses (Muller and Scott, 1981). Even though this indirect coupling led in our contralateral double recordings to a substantial increase in EPSPs and IPSPs compared to the unstimulated conditions, the effect was significantly smaller than in ipsilateral T cell pairs. Consistently with the expectation for polysynaptic connections, only few spikes were triggered with a very long latency of several hundred of milliseconds after the onset of the contralateral presynaptic current injection (Figures 5 G, H).

In conclusion, T cells at both soma locations seem to receive postsynaptic potentials of similar heights and duration from the network and mutually from each other. Nevertheless, this does not imply identical synaptic partners. Anatomical studies showed that T cells share gap junctions with approximately 30 other neurons (Segura et al., 2020) and contain pre- and postsynaptic structures in close proximity on their cell processes (Muller and McMahan, 1976), like most neuronal arbors in the leech neuropil (Pipkin et al., 2016). Hence, the recorded synaptic potentials could stem from different presynaptic partners, which in total influence the T cell at both locations in a similar way. The overlapping, but not identical set of Neurobiotin-coupled neurons, that can be seen in the examples in Figure 6, fit well into the spectrum found by Segura et al. (2020), and could support this idea. However, diverse network interactions are not specific to different soma locations or anatomical subtypes, but even apply to the unequivocally defined T cells with one root process and a dorsal receptive field (Segura et al., 2020). In conclusion, the inter-cell variations in postsynaptic responses could be an indication of a continuum of circuit-level set points across animals, which all lead to robust function despite different network connections, like it was proposed for stellate cells in mouse medial entorhinal cortex (Pastoll et al., 2020).

### 4.3. Effects of anatomy

In contrast to the time-dependent changes in excitability and synaptic inputs, which seem to form a continuum rather than two distinct groups, our anatomical studies revealed a clear difference between the T cells at soma location T2 vs. T3. In our sample of 18 T2 cells, all cells innervated the skin via two root processes. In contrast, the sample of 46 T3 cells fell into two equally probable anatomical categories, as well as the 11 filled cells at location T1. That does not imply that T cells at T2 never have only one branch, as can be seen from previously published examples of one-branched cells at soma location T2 (Nicholls and Baylor, 1968; Kretzberg et al., 2016). Still, our sample indicates clearly that the dorsal T cell has a higher probability to be located at soma location T1 or T3 than at T2.

Our findings confirm the observation that cell body locations and other anatomical details vary between ganglia (Pipkin et al., 2016). It also explains that the T cell subtype with only one root branch innervating the dorsal receptive field was found at location T3 according to Blackshaw (1981), but at T1 according to Kretzberg et al. (2016). A clear example of this variation is presented in Figure 6E, where one T cell at soma location T1 has one root process and therefore presumably a dorsal receptive field, while its contralateral partner at T1 has two root processes. Since this variation occurs between cells even in the same ganglion in the same animal, it seems not to be genetically predetermined, but ontogenetically influenced.

The striking finding that the approximately 50% of T3 cells with only one root process were significantly less excitable than the T cells with two root processes (at the soma locations T3 as well as T2) suggests a causal relationship. T cells that stretch their processes via two roots to the skin have a larger surface and provide probably more space for spike-generating voltage-gated ion channels. On the other hand, smaller cells tend to have a higher input resistance, which can result in a higher spike count, because the passive response to current injection depolarizes the membrane potential further beyond the spike threshold (Kernell, 1966). It should be kept in mind that our recordings were performed in the somata of spatially extended cells. Our input resistance measurements are affected mainly by the membrane close to the soma, while spikes could be generated distant from the soma. Therefore, a high somatic input resistance might not directly lead to many spikes. We found that the distributions of recorded input resistances were broad and highly overlapping for both soma locations. The sample of T2 cells had a (not statistically significant) tendency toward a higher input resistance than the T3 cells (Figure 2D), even though the T2 cell sample probably contained a higher percentage of T cells with two root processes. As test for a causal relationship between the anatomy and the excitability, we performed compartmental modeling with identical sets of biophysical parameters applied to different anatomical structures. Our simulations of reconstructed T3 neurons with one vs. two root processes reproduced the main neuronal response features and yielded the experimentally observed broad distributions of input resistances. These simulations with identical homogeneous channel densities did not predict a difference in excitability between the two anatomical classes, ruling out a causal relationship (Figure 7). Nevertheless, it should be noted that our simulations were all based on neurons with soma location T3. Since it was shown that the location of the soma in invertebrate neurons impacts signal attenuation (Hesse and Schreiber, 2015), we cannot exclude that the soma location T2 would lead to systematic differences in excitability.

### 4.4. Outlook

After excluding the coarse anatomical structure of the T cells with one vs. two root branches as the sole reason for the significant differences in excitability, future studies need to focus on the ensemble of ion channel types and their spatial distribution over the neuronal compartments. The distribution of ion channels in the membrane was shown to vary between different cortical and hippocampal cell types and thereby to increase neuronal diversity (Nusser, 2009). For cortical neurons, compartmental modeling showed that the location of ion channels in the membrane impacts the shape and number of action potentials (Brette, 2013). Compared to a vertebrate neuron, that integrates dendritic inputs at the soma and generates action potentials at the axon hillock (Thome et al., 2014), the location of spike generation is less stereotypic in invertebrate neurons (Smarandache-Wellmann, 2016). For leech annulus erector motor neurons (AE), laser ablation experiments revealed that spikes are generated at the primary bifurcation point of the neurite, located several 100 μm away from the passive soma, leading to very small spike amplitudes in somatic recordings (Gu et al., 1991). T cells were shown to have at least two spatially separated spike-initiation zones, one in the periphery that conveys information about touch stimuli, and a central one in the ganglion that responds to synaptic inputs (Burgin and Szczupak, 2003; Kretzberg et al., 2007). If central spike initiation in T cells took place in the root processes or their branching point from the central neurite, ventral and lateral T cells with two root processes might have two central spike initiation zones and therefore be more excitable by somatic current injection. Even though our current simulations were already performed with compartmental models of reconstructed individual cell anatomies, these were limited to a homogeneous channel distribution. Future simulations with inhomogeneous channel distributions will allow the systematic variation of channel densities in the membrane of different compartments and might provide insights into the basis of the observed diversity of electrophysiological phenotypes of the same cell type.

Finally, future studies need to address biophysical diversity by addressing the ion channel types and their covariation across cells. The across-cell variability in electrophysiological responses could come from different ion channel properties (Johansen, 1991; Kleinhaus and Angstadt, 1995). Deep sequencing of mouse somatosensory dorsal root ganglion neurons revealed different expression patterns of functionally relevant genes between neuronal subtypes and corresponding electrophysiological response (Zheng et al., 2019). The considerable variability in gene expression of brainstem neurons was interpreted as a phenotype gradient associated with cellular input history that supports robust biological function (Park et al., 2014). Sequencing studies of leech T cells neither distinguished the T cells by receptive field, nor by soma location. However, on a cluster analysis of gene expression profiles, the three T cells constituted a tight cluster that was clearly separated from other cell types, e.g., by a distinct IP3 receptor, while still showing some variability between each other (Heath-Heckman et al., 2021). A study of the leech genome identified 40 K^+^, 4 Na^+^, 10 Ca^2+^, and 5 TRP channel contigs (Northcutt et al., 2018). Transcriptional profiling of identified leech cell types revealed their distinctive transcriptional profiles. The covariation in expression could lead to a whole range of channel conductance and kinetics, as well as to different ion channel distribution in the membrane. Hence, the broad distributions of all response properties within the same cell type might be a result of the covariation in expression of functionally overlapping ion channels (Goaillard and Marder, 2021). This leaves us with the question how the biophysical and spatial channel composition shapes neuronal responses and leads to variability within and systematic differences between anatomical (sub)types of cells. Experimentally, patch clamp and immunofluorescence staining are needed to understand the range of T cell phenotypes. These experimental results will provide the basis for systematic parameter variations of the biophysical features and inhomogeneous spatial distribution of ion channels in the anatomically detailed compartmental models of individual reconstructed cells. Combined experimental and modeling studies will shed light on the question if the observed electrophysiological variability is the result of a combination of continuous distributions of channel expressions and locations, or if separated clusters of parameters indicate sub-types. Each of the six T cells in a ganglion is the unique encoder of the light touch in its receptive field. Therefore, this approach might be the key to understanding the diversity of solutions with which a nervous system that is too small for redundancy can react robustly to sensory stimulation. Hence, the well-accessible leech nervous system is highly suitable for further combined experimental and modeling studies to tackle the fundamental question of neuronal diversity, which is relevant far beyond the leech.

## Data availability statement

The datasets presented in this study can be found in online repositories. The names of the repository/repositories and accession number(s) can be found below: https://gin.g-node.org/wuso3118/Meiser_et_al_2023/.

## Author contributions

SM and JK: conceptualization, interpretation, and drafting manuscript. JS: electrophysiological data collection (Figures 1–5). SM and IA: anatomical studies (Figure 6). KS: modeling (Figure 7). JK: project administration and supervision. All authors contributed to the article, including data analysis and figures design, and approved the submitted version.
