# Supplementary material for: Cell anatomy and network input explain differences within but not between leech touch cells at two different locations
Source: Front Cell Neurosci. 2023 Jul 25;17:1186997. doi: 10.3389/fncel.2023.1186997 (PMC10411907; doi:10.3389/fncel.2023.1186997)
Supplement: Supplementary file 1 [file Table_1.pdf]

Supplementary Table 1

Number of Leeches, Ganglia, and Neurons included in each of the data sets. Figures 2 – 5 analyze subsets of the electrophysiological double recordings, and Figures 6 and 7 display examples of anatomical studies.

Data Set	Leeches	Ganglia	Neurons
**Total number of electrophysiological double recordings**	*60*	*82*	*164*
Figure 2: Physiological parameter distribution for T2 and T3 cells in response to the test protocol	*60*	*79*	*129*
Figure 3: Comparison of T2 and T3 cell spike shapes	*60*	*79*	*112*
Figure 4: Comparison of SC and RMP changes over time in T2 and T3 cells.	*60*	*82*	*164*
Figure 5: Spontaneous network input and mutual interaction of T2 and T3 cells	*19*	*42*	*84*
**Total number of anatomical studies**	*30*	*35*	*76*
Figure 6: Neurobiotin fills to study anatomies with one versus two root processes (4 examples shown in the figure)	*29*	*34*	*75*
Figure 6: Autofluorescence ganglion map (Alexa filled interneuron)	*1*	*1*	*1*
Figure 7: 3D Reconstruction / Modelling	*10*	*17*	*17*
