# Supplementary material for: Cell anatomy and network input explain differences within but not between leech touch cells at two different locations
Source: Front Cell Neurosci. 2023 Jul 25;17:1186997. doi: 10.3389/fncel.2023.1186997 (PMC10411907; doi:10.3389/fncel.2023.1186997)
Supplement: Supplementary file 2 [file Table_2.pdf]

Supplementary Table 2

Statistical test results of T2 and T3 cell response features. Statistically significant effects are marked in bold. p, statistical p-value; z, value of the z-statistics; n1/n2, number of the cells within the x or y variable.

	**Wilcoxon tests**					
	x	**y**	**n1/n2**	**z**	**p**	**Paired/unpaired**
**Figure 2: Physiological parameter distribution for T2 and T3 cells in response to the test protocol,** *α= 0.05*	Initial SC T3	Initial SC T2	68/61	5.3692	**7.9068e-08**	unpaired
	Initial LAT T3	Initial LAT T2	68/61	-4.7239	**2.3132e-06**	unpaired
	Initial RB SC T3	Initial RB SC T2	68/61	2.4618	**0.0138**	unpaired
	Initial IR T3	Initial IR T2	68/61	1.1959	0.2318	unpaired
	Initial RMP T3	Initial RMP T2	68/61	1.4129	0.1577	unpaired
**Figure 3: Comparison of T2 and T3 cell spike shapes,** *α = 0.05*	Second spike amplitude T3	Second spike amplitude T2	54/58	3.3199	**9.0045e-04**	unpaired
**Figure 4: Comparison of SC and RMP changes over time in T2 and T3 cells.** *Correction for multiple testing with Bonferroni (α = 0.0125)*	Initial SC stim T3 – 18s	0	20	2.1102	0.9863	Paired
	Initial SC stim T3 – 318s	0	20	1.5096	0.0656	Paired
	Initial SC stim T3 – 618s	0	19	3.1031	**0.0010**	Paired
	Initial SC stim T3 – 918s	0	20	3.5491	**0.0002**	Paired
	Initial RMP stim T3 – 18s	0	20	-0.5413	0.2941	Paired
	Initial RMP stim T 3 – 318s	0	20	-0.8400	0.2005	Paired
	Initial RMP stim T3 – 618s	0	19	-2.8186	**0.0043**	Paired
	Initial RMP stim T3 – 918s	0	20	-1.2337	**0.0108**	Paired
	Δ SC stim T3 – 18s	0	20	2.3622	**0.0091**	Paired
	Δ SC stim T3 – 318s	0	20	1.7647	0.0388	Paired
	Δ SC stim T3 – 618s	0	19	1.2266	0.1100	Paired
	Δ SC stim T3 – 918s	0	20	1.10276	0.1351	Paired
	Δ RMP stim T3 – 18s	0	20	-1.5120	0.0653	Paired
	Δ RMP stim T3 – 318s	0	20	0.9839	0.8387	Paired
	Δ RMP stim T3 – 618s	0	19	0.1948	0.5772	Paired
	Δ RMP stim T3 – 918s	0	20	-1.1386	0.1274	Paired
	Initial SC stim T2 – 18s	0	20	0.68571	0.2464	Paired
	Initial SC stim T2 – 318s	0	20	3.1618	**0.0008**	Paired
	Initial SC stim T2 – 618s	0	20	3.3411	**0.0004**	Paired
	Initial SC stim T2 – 918s	0	23	2.8531	**0.0022**	Paired
	Initial RMP stim T2 – 18s	0	20	-1.8480	0.0323	Paired
	Initial RMP stim T2 – 318s	0	20	-0.8400	0.0229	Paired
	Initial RMP stim T2 – 618s	0	20	-2.6297	**0.0024**	Paired
	Initial RMP stim T2 – 918s	0	23	-2.2960	0.1087	Paired
	Δ SC stim T2 – 18s	0	20	3.48589	**0.0002**	Paired
	Δ SC stim T2 – 318s	0	20	2.54350	**0.0055**	Paired
	Δ SC stim T2 – 618s	0	20	1.8761	0.0303	Paired
	Δ SC stim T2 – 918s	0	23	0.5449	0.7071	Paired
	Δ RMP stim T2 – 18s	0	20	-1.4373	0.0753	Paired
	Δ RMP stim T2 – 318s	0	20	-0.6533	0.2568	Paired
	Δ RMP stim T2 – 618s	0	20	-2.8186	0.0024	paired
	Δ RMP stim T2 – 918s	0	23	-1.0064	0.1571	paired
	Initial SC stim T2 – 18s	Initial SC stim T3 – 18s	20/20	-1.9230	0.0545	unpaired
	Initial SC stim T2 – 318s	Initial SC stim T3 – 318s	20/20	-3.6179	**2.9699e-04**	unpaired
	Initial SC stim T2 – 618s	Initial SC stim T3 – 618s	20/19	-1.5503	0.1211	unpaired
	Initial SC stim T2 – 918s	Initial SC stim T3 – 918s	23/20	-0.3048	0.7605	unpaired
	Initial RMP stim T2 – 18s	Initial RMP stim T3 – 18s	20/20	0.2287	1.2037	unpaired
	Initial RMP stim T2 – 318s	Initial RMP stim T3 – 318s	20/20	0.3793	0.8791	unpaired
	Initial RMP stim T2 – 618s	Initial RMP stim T3 – 618s	20/19	1.7897	0.0735	unpaired
	Initial RMP stim T2 – 918s	Initial RMP stim T3 – 918s	23/20	-0.7427	0.4577	unpaired
	Δ SC stim T2 – 18s	Δ SC stim T3 – 18s	20/20	-0.5545	0.5792	unpaired
	Δ SC stim T2 – 318s	Δ SC stim T3 – 318s	20/20	1.2578	0.2085	unpaired
	Δ SC stim T2 – 618s	Δ SC stim T3 – 618s	20/19	2.7393	**0.0062**	unpaired
	Δ SC stim T2 – 918s	Δ SC stim T3 – 918s	23/20	0.6488	0.5164	unpaired
	Δ RMP stim T2 – 18s	Δ RMP stim T3 – 18s	20/20	-0.5545	0.5792	unpaired
	Δ RMP stim T2 – 318s	Δ RMP stim T3 – 318s	20/20	1.2578	0.2085	unpaired
	Δ RMP stim T2 – 618s	Δ RMP stim T3 – 618s	20/19	2.7393	**0.0062**	unpaired
	Δ RMP stim T2 – 918s	Δ RMP stim T3 – 918s	23/20	0.4992	0.6177	unpaired
**Figure 5: T2 and T3 cells receive spontaneous network input and react to presynaptic T cell stimulation with postsynaptic potentials and spikes.**	Spontaneous IPSPs T2	Spontaneous IPSPs T3	42/42	1.3464	0.1782	unpaired
	Spontaneous EPSPs T2	Spontaneous EPSPs T3	42/42	0.5502	0.5822	unpaired
	Spontaneous spikes T3	Spontaneous spikes T2	42/42	0.7554	0.4500	unpaired
	IPSPs T2 contra stim	IPSPs T3 contra stim	23/19	0.4963	0.6197	unpaired
	EPSPs T2 contra stim	EPSPs T3 contra stim	23/19	0.9050	0.3654	unpaired
	IPSPs T3 ipsi stim	IPSPs T2 ipsi stim	23/23	-0.8129	0.4163	unpaired
	EPSPs T3 ipsi stim	EPSPs T2 ipsi stim	23/23	-0.1758	0.8605	unpaired
	IPSPs T3 contra stim	IPSPs T3 ipsi stim	19/23	2.5523	**0.0107**	unpaired

Correction for multiple testing with Bonferroni (α = 0.0167)	EPSPs T3 contra stim	EPSPs T3 ipsi stim	19/23	3.2599	**0.0011**	unpaired
	IPSPs T2 contra stim	IPSPs T2 ipsi stim	19/23	0.5560	0.5782	unpaired
	EPSPs T2 contra stim	EPSPs T2 ipsi stim	19/23	1.9458	0.0517	unpaired
	Spontaneous IPSPs T3 contra	IPSPs T3 contra stim	19/19	-2.0436	0.0410	paired
	Spontaneous IPSPs T3 ipsi	IPSPs T3 ipsi stim	23/23	-4.0863	**4.3837e-05**	paired
	Spontaneous IPSPs T2 contra	IPSPs T2 contra stim	19/19	-2.9219	**0.0035**	paired
	Spontaneous IPSPs T2 ipsi	IPSPs T2 ipsi stim	23/23	-3.9997	**6.3428e-05**	paired
	Spontaneous EPSPs T3 contra	EPSPs T3 contra stim	19/19	-3.2114	**0.0013**	paired
	Spontaneous EPSPs T3 ipsi	EPSPs T3 ipsi stim	23/23	-5.5582	**2.7257e-08**	paired
	Spontaneous EPSPs T2 contra	EPSPs T2 contra stim	19/19	-4.4668	**7.9400e-06**	paired
	Spontaneous EPSPs T2 ipsi	EPSPs T2 ipsi stim	23/23	-4.7234	**2.3196e-06**	paired
	Latency T3 ipsilaterale	Latency T2 ipsilaterale	23/23	0.6982	**0.4851**	unpaired
	T2 Spikes Stim contra	T2 Spikes Stim ipsi	19/19	-3.4287	**6.0659e-04**	unpaired
	T3 Spikes Stim_contra	T3_Spikes Stim ipsi	23/23	-2.7088	**0.0068**	unpaired
	T2 Spikes Stim contra	T3 Spikes Stim contra	19/19	-0.4312	0.6663	unpaired
	T2 Spikes Stim_ipsi	T3_Spikes_Stim_ipsi	23/23	-0.2581	0.7963	unpaired
	Spontaneous spikes T3 contra	T3 Spikes Stim contra	19/19	-1.7948	0.0727	paired
	Spontaneous spikes T3 ipsi	T3 Spikes Stim ipsi	23/23	-3.5662	**3.6219e-04**	paired
	Spontaneous spikes T2 contra	T2 Spikes Stim contra	19/19	-3.5662	0.3948	paired
	Spontaneous spikes T2 ipsi	T2 Spikes Stim ipsi	23/23	-4.0338	**5.4878e-05**	paired
